# Estimation of Machining Sustainability Using Fuzzy Rule-Based System

**DOI:** 10.3390/ma14195473

**Published:** 2021-09-22

**Authors:** Asif Iqbal, Guolong Zhao, Quentin Cheok, Ning He

**Affiliations:** 1 Faculty of Integrated Technologies, Universiti Brunei Darussalam, Jalan Tungku Link, Gadong BE 1410, Brunei; quentin.cheok@ubd.edu.bn; 2 College of Mechanical & Electrical Engineering, Nanjing University of Aeronautics & Astronautics, 29-Yu Dao Street, Nanjing 210016, China; zhaogl@nuaa.edu.cn (G.Z.); drnhe@nuaa.edu.cn (N.H.)

**Keywords:** cutting, fuzzy sets, fuzzy reasoning, sustainable machining, cutting fluid

## Abstract

Quantification of a highly qualitative term ‘sustainability’, especially from the perspective of manufacturing, is a contemporary issue. An inference mechanism, based on approximate reasoning, is required to tackle the complexities and uncertainties of the manufacturing domain. The work presents development of a fuzzy rule-based system to quantify sustainability of the most widely utilized manufacturing process: machining. The system incorporates the effects of key control parameters of machining on several sustainability measures, as reported in the literature. The measures are categorized under the three dimensions of sustainability and contribute to the sustainability scores of the respective dimensions with different weightages. The dimensions’ scores are added up in different proportions to obtain the holistic sustainability score of the process. The categories of the control parameters incorporated into the system include type of the process, work material, material hardness, tool substrate and coating, tool geometry, cutting fluids, and cutting parameters. The proposed method yields sustainability scores, ranging between 0 and 100 of machining processes against the given values of their prominent control parameters. Finally, the rule-based system is applied to three different machining processes to obtain the measures of their accomplishment levels regarding economic, environmental, and societal dimensions of sustainability. The sustainability score of each process is then obtained by summing up the three accomplishment levels under the respective weightages of the dimensions. The presented approach holds immense potentials of industrial application as it can conveniently indicate the current sustainability level of a manufacturing process, leading the practitioners to decide on its continuation or improvement.

## 1. Introduction

The issues concerning environmental degradation, such as emissions of greenhouse gases, landfilling, global warming, water pollution, and depletion of natural resources have gained massive attention during the last couple of decades. The planet’s deterioration is rightly pinned on the human development and, thus, for the remedy, a merited call for moving on from ‘reckless development’ to ‘responsible development’ is being raised. Manufacturing has always remained a key ingredient of human development and, therefore, needs to be revamped under the sustainability principles. The manufacturing realm, comprising systems, technologies, and processes, need to be sustainable rather than profit-mongering for reassurance of the planetary resources and passing on the benefits of human development to the future generations.

Sustainability, in respect of manufacturing, is being profoundly researched and discussed but, similar to other domains, is still considered as a qualitative attribute [1]. To effectively improve the manufacturing domain, methods need to be worked out to quantify sustainability. Unfortunately, modeling of a manufacturing process or system in its entirety is a highly complex task that involves a great deal of vagueness in the variables and uncertainties in the values and events. Furthermore, quantification of a qualitative attribute (sustainability) in respect of a highly complex realm (manufacturing) becomes more intricate. As analytical modeling is rendered impossible, approximate reasoning becomes a fitting approach for quantifying manufacturing sustainability.

Sustainability stands on three pillars: economy, environment, and society [2]. The quantification process requires the performance measures of a manufacturing process or system to be related to each of the three pillars (dimensions) on one hand and mapped to the major control parameters on the other. The mapping of the performance measures with the control parameters is based on the effects of the latter on the former, which are determined through the experimental data and represented in the form of fuzzy statements. Fuzzy reasoning is needed to keep the mapping generalized and all-inclusive in respect of enormity of the process or the system. Machining, the most extensively practiced manufacturing process, is explored in this work to actualize the approach of sustainability estimation. Machining is defined as a subtractive manufacturing process in which the desired shape of the part is achieved by successively removing the material from the part’s surface in form of a chip [3]. The performance of this manufacturing process is characterized by the measures of material removal rate, tool life and acquisition cost, cutting forces and specific cutting energy, dimensional accuracy and work surface roughness, and waste generation. These performance measures contribute toward the three dimensions of sustainability with different roles and weightages. A brief review of the literature focusing on the application of knowledge-based systems in quantification of manufacturing sustainability is further provided:

Alblas et al. [4] have reported that sustainability demands, and incentives can be fuzzy or even absent in the context of a product-making firm. The authors have emphasized that the addressal of fuzziness in sustainability incentives is crucial for meaningful implementation of sustainability tools in the context of new product development. Giovannini et al. [5] have applied product-driven ontology for structuring knowledge of sustainable manufacturing. The authors have developed a knowledge-based system for generating machine codes from the product’s specification under the requirement of implementing sustainability in product design and process planning. A multigrade fuzzy method is used to evaluate Environmental Sustainability Index in respect of an automotive manufacturing industry [6]. Ocampo has applied fuzzy analytic hierarchy process to identify the content of a sustainable manufacturing strategy [7]. Linguistic variables along with triangular fuzzy numbers were utilized to explicate reasoning of the elements. A combination of fuzzy inference system and fuzzy analytical hierarchal process is used to evaluate sustainability of manufacturing SMEs (small and medium-sized entreprises) [8]. The approach is claimed to be useful in assessing effectiveness of a sustainability strategy when dealing with suppliers from SMEs. Yadegaridehkordi et al. [9] have introduced Green Building Index as a sustainability rating tool in green building manufacturing sector. Likewise, Rezvan et al. [10] have presented a hybrid approach of analytical hierarchy process and fuzzy inference system to quantify sustainability of concrete manufacturing process. The authors have incorporated all the three dimensions of sustainability in the computations, which are based on the principles of Life Cycle Assessment. Belkadi et al. [11] have proposed an integration of knowledge-based systems and product life cycle management for optimizing resource consumption in a manufacturing setup. The authors have emphasized usage of the integrated sustainability tool in production planning for minimization of resource consumption at a given level of productivity. A study has focused on modeling product recovery strategy through the attributes of waste, time, and cost of a manufactured product for the sake of reducing landfilling waste [12]. Choudhry et al. have developed a three-level hierarchical model by classifying key performance indices into sustainability features and vital supply chain decision-making areas [13]. Ahmad et al. have proposed an integrated sustainability assessment approach that combines fuzzy and stochastic uncertainties [14]. The approach has allowed the authors to include both qualitative and quantitative, and weighted sustainability indicators to assess sustainability level of a food manufacturing firm. Halfdanarson and Kvadsheim have provided a way forward for manufacturing companies which are seeking knowledge to transform their operations to sustainability-rich activities supportive of circular economy [15]. The social dimension of sustainability in the context of manufacturing is often overlooked [16]. The authors have presented a framework for assessing social sustainability from the viewpoint of ergonomics. The three aspects included in the structure are work environment, man-machine interaction, and work task. Likewise, Rajak and Vinod have proposed an estimation approach for quantifying social sustainability performance of a manufacturing-intensive organization [17]. The study also provides a mechanism for computing fuzzy Social Sustainability Index.

Fuzzy reasoning and knowledge-based systems have found applications in sustainable and green machining as well. A sustainability estimation model is presented that incorporates green features of a machining process in three aspects: economy, productivity, and environmental impact [18]. The authors have employed a multi-grade fuzzy approach to develop the sustainability estimation model. Iqbal et al. [19] have developed two fuzzy expert system-based approaches for the estimation of tool damage in a hard milling process. The approach incorporating real-time cutting force signals is reported to have outperformed the one utilizing length of cut as the indicator of the tool wear’s state. Deng et al. [20] have presented an expert system for optimizing green cutting process. A specific emphasis is placed on estimating cutting tool’s energy consumption. For its sake, a mathematical model is developed from experimental data, and is connected with the expert system’s rule-base. Xu et al. [21] have presented an intelligent reasoning system that estimates specific cutting energy consumption at various tool wear states of a milling process for the sake of optimizing the cutting parameters. Iqbal et al. [22] have presented working of a self-developing fuzzy expert system aimed at optimizing the control parameters against a set of objective functions and predicting the performance measures at the optimized settings of the parameters. The expert system possesses the capabilities of self-learning, self-correction, and self-expansion. Grinek et al. [23] have developed fuzzy models for optimizing cutting speed in a machining process based on temperature dependent strength of the work material.

The review of the relevant literature provided above suggests that knowledge-based systems and fuzzy logic have found their ways to assess and quantify sustainability of the different aspects of manufacturing. However, the application remains limited in scope as either all the dimensions of sustainability are not addressed, or a process/system is not covered comprehensively and holistically. Most of the investigations remain focused on either assessing sustainability of particular factories, rather than technologies/processes, or improving sustainability of support systems, such as production planning and supply chain management. Furthermore, the articles undertaking a particular manufacturing technology, such as machining, have considered only a particular class of control parameters for the sake of sustainability assessment. In this context, a research gap needs to be filled by considering all the three dimensions of sustainability and including all the influential control parameters of a manufacturing process for estimation of its sustainability. As such, an all-inclusive approach rules out analytical modeling in respect of a complex physical process, the approximations actualized by fuzzy reasoning are obviously productive. In this regard, the current work presents development of a fuzzy reasoning system, comprising IF-THEN rules, to estimate a sustainability score (SS) of machining processes by incorporating effects of all the significant parameters on the three dimensions of sustainability: economy, environment, and society.

## 2. Materials and Methods

### 2.1. The Process and its Sustainability Measures

The manufacturing process chosen to epitomize the sustainability assessment approach in this work is machining. Machining is a subtractive manufacturing process in which the desired shape of a part is achieved by successively removing the material from the part’s surface in form of a chip. Due to its strengths regarding geometric precision, surface finish, shape versatility, and a range of workable materials, machining is rightfully the most widespread manufacturing technology in the world [3]. Although machining is a direct contributor toward national economy on one hand, as it transforms the natural resources into GDP, it also impacts the environment negatively on the other. The technology is known to degrade the environment in a number of ways, such as consumption of electrical energy that leads to generation of more energy and subsequent emission of greenhouse gases, generation of processing waste that needs cleaning, recycling, and landfilling, depletion of natural resources in form of scarce metals which are used in making of cutting tools, and pollution of land and water through disposal of cutting fluids.

Prior to promulgation of the sustainability requirements and regulations, the machining domain was assessed in terms of performance measures, comprising of economy-oriented metrics, such as tool life, material removal rate, process cost, dimensional accuracy, and work surface quality. The global ratification of the sustainability norms has supplanted the list of “performance measures” with that of “sustainability measures”. The latter includes additional metrics, such as specific cutting energy, swarf generation, personal safety, and more.

Considering the contemporary levels of prominence being received by the three dimensions of sustainability in respect of manufacturing industry, it is rational to assign economy, environment, and society the weightages of 40%, 40%, and 20%, respectively in the calculation of the sustainability score (SS). Pragmatically, societal measures, such as working hours, health, safety, and others collectively hold more or less half of the significance as that of economy or environment. Furthermore, the control parameters of most of the modern-day manufacturing technologies, including machining, do not affect the societal measures as significantly as they do the others. The main equation for the sustainability score, in respect of a machining process, can, thus, be written as follows:

*SS* = 0.4(*SS*_eco_ + *SS*_env_) + 0.2 *SS*_soc_ (1)

where *SS*_eco_, *SS*_env_, and *SS*_soc_ represent the individual sustainability scores of the dimensions of economy, environment, and society, respectively.

Undoubtedly, the values of these weightages can be changed according to any specific preference. The variation in these numbers does not change the quantification approach by any means. Obviously, the sustainability score would be changed, resulting in a better quantification result from the perspective of that preference. Sustainability score, as discussed in this work, is an approximate evaluation of a process’s performance in the context of sustainability. Industrial decisions on whether to uphold a process or revamp it for an improvement in the sustainability (or any of its dimensions) do not need very precise numbers on sustainability scores.

The sustainability measures of a machining process can be categorized according to the dimensions of sustainability they contribute toward. Table 1 presents the lists of the measures grouped under each of the three sustainability dimensions along with the respective percentage shares toward that dimension. Six measures are listed under economy. Tool acquisition economy is a measure of purchase cost of the tooling involved. Its value depends on the tooling substrate, coating, and geometric dimensions. The higher is the purchase cost the lower is the value of this measure. Tool life is defined as the volume of work material removed by a given tool just prior to fulfillment of the tool replacement criterion. The most commonly applied criterion is the growth of the width of the flank wear land beyond a specific value. The longer is the tool life the better is it for economic sustainability. The two aforementioned sustainability measures stand in opposition to each other. In other words, a machining tool with a high acquisition economy is expected to yield a relatively short tool life.

Productivity describes the volume of output delivered per unit of a resource consumed. The resource, in respect of manufacturing processes, could be processing time (machine-hours/manhours) or energy/material consumed. The most relevant productivity metric for a subtractive manufacturing process is the volume of work material removed per unit time. In machining terms, the metric is named as material removal rate (MRR) and quantified in the unit of mm^3^/s. Economic sustainability seeks a high value of this measure. Fluid consumption economy is related to the cost of supplying a cutting fluid for providing lubrication and cooling effect during the cutting process. A dry cutting process yields the highest value of this measure. On the other hand, costly fluids, such as cryogenic coolants, return low values. Surface quality is another important economic measure of sustainability. In machining, the measure governs whether a machined part would be passed for the next steps of production or scrapped or reworked. A scrapped part represents the lowest level of economic sustainability as the disposition causes wastages of the work material as well as the energy consumed. A rework disposition is better, yet not the best, as it salvages the work material but not the energy consumed. Electricity conservation is a measure related to the cost of the primary energy usage per unit volume of the work material removed. The higher the specific energy consumption the lower is the value of the measure. The percentage shares assigned to the measures of economic sustainability, as shown in Table 1, are in accordance with their respective contributions toward total machining cost as observed in the present-day machining industry [24].

Specific energy saving, an environmental sustainability measure, is inverse of specific energy consumption. Its high value (favorable for environmental benignity) indicates that a low level of energy is consumed in removing a given volume of work material. Swarf avoidance stands for cleanliness in chip forming. Its high value is an indicator of oil/emulsion free, easily collectable chips, which helps to preclude an energy- and material-intensive requirement of chips cleaning prior to initiating the recycling process. Landfill avoidance (tool) and landfill avoidance (workpiece), respectively, are measures which indicate the volume of work material removed before a cutting tool and a workpiece are disposed as scrap. A high value of the former indicates a long tool life whereas that of the latter indicates a high surface quality of the machined surface that averts non-conformance to the quality specification and subsequent scrapping of the part. The respective share assigned to each of the four measures, under the category of environmental sustainability, is based on the relative and approximate impact caused on the environment. With regard to the societal measures of sustainability, health and safety are self-explanatory. The third measure, working hours, is related to productivity. Understandably, a higher MRR would cause the machine operators to spend lesser time in the working area against a given production target. Among the three societal measures, safety is given a slightly higher weightage than the other two, as shown in Table 1.

#### The Control Parameters

In order to quantify machining sustainability, it is pertinent to depict the effects of the major machining control parameters on the sustainability measures discussed beforehand. A bulk of literature, based on experimental investigations, is available that has established the true effects of the machining predictors on the various responses. The findings of the published work can be aggregated to find general effects of the control parameters on the sustainability measures. The major machining parameters mapped in this work can be grouped into the following seven categories: (a) machining type (turning /milling/drilling), (b) work material (carbon steel/alloy steel/titanium alloy), (c) mechanical property of the material (surface hardness), (d) tool material (high-speed steel/carbide/coated carbide/polycrystalline cubic boron nitride), (e) tool geometry (rake angle/helix angle/drill diameter), (f) cutting fluids (dry/emulsion/minimum quantity of lubrication/air/liquid nitrogen/compressed carbon dioxide), and (g) cutting parameters (cutting speed/feed rate/depth of cut). The parameters grouped under the categories (c), (e), and (g) are numeric whereas the others are categorical.

The experimental findings, regarding the effects of the aforementioned machining parameters on the sustainability measures, published in over 115 papers, dissertations, and book chapters were collected, organized, correlated, and reviewed to establish their generalized impacts on the 13 metrics listed in Table 1. Clearly, the “generalized impacts” do not manifest precise quantifications of the “effects” but are good enough to constitute a system for estimation of an indistinct metric, such as sustainability. The next section presents a mechanism to amalgamate the generalized impacts of the process parameters to obtain a holistic system of predicting sustainability.

## 3. The Fuzzy Rule-Base

The knowledge condensed from the published reports needs to be expressed in the form of IF-THEN rules. Prior to that, a technique needs to be adopted to tackle inherent impreciseness in the data and vagueness of the terms. Fuzzy reasoning stands up to the situation that can effectively cope with the issues of impreciseness and vagueness. For its implementation, fuzzy sets are developed for all the numeric machining control parameters and percentage shares of the sustainability measures and. Therefore, fuzzified data in respect of the numeric variables will be used in working of the rules.

### 3.1. Fuzzy Sets

**Figure 1:**
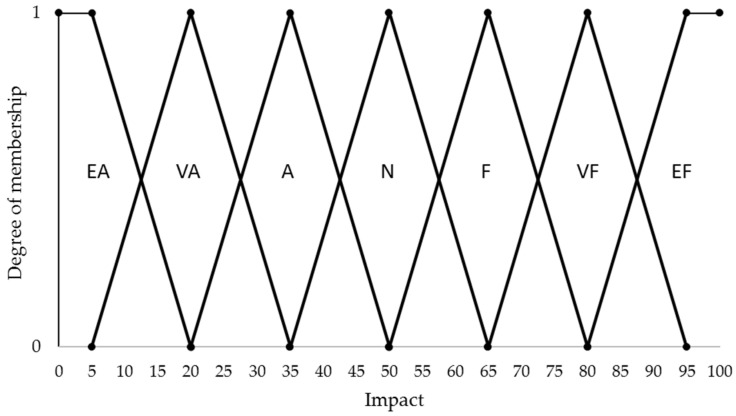
Fuzzy set for the impact of a machining control parameter on a sustainability measure.

Figure 1 presents the fuzzy set for the impact of a machining control parameter on a sustainability measure. Seven equally distributed membership functions cover the range from 0 to 100. The members’ labels EA, VA, A, N, F, VF, and EF, respectively stand for extremely adverse, very adverse, adverse, neutral, favorable, very favorable, and extremely favorable. The set is common for the 13 sustainability measures listed in Table 1. Its members describe the role and strength of the parameter’s effect on the sustainability measure. A 50–100 range of impact represents a favorable role of the control parameter in respect of the sustainability measure with the high values symbolizing high strengths. Likewise, a 0–50 range describes an adverse role with the low values indicating strong effects.

**Figure 2:**
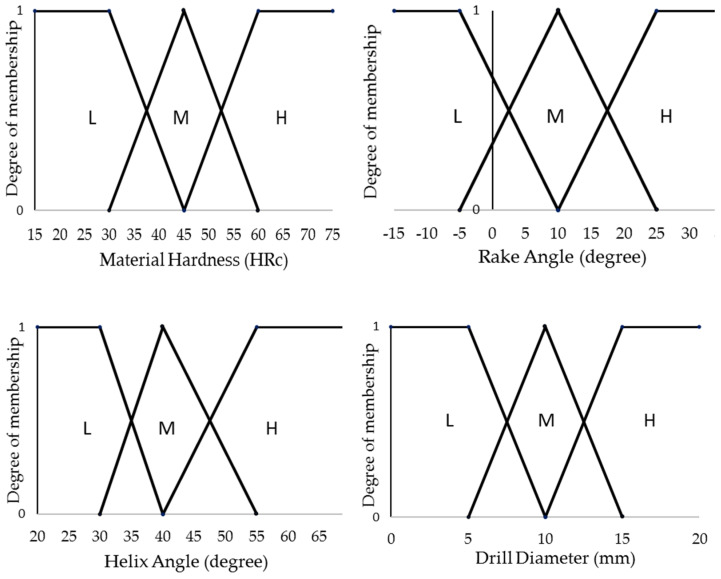

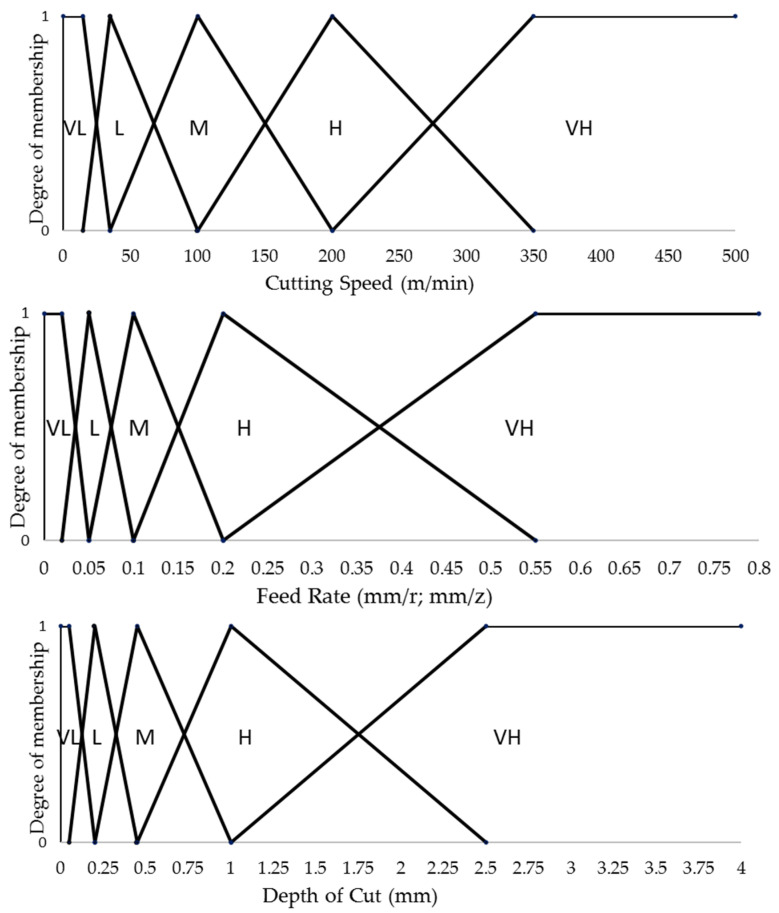
Fuzzy sets for the numeric control parameters of machining.

Figure 2 presents the triangular fuzzy sets for all the numeric control parameters, namely work surface hardness, rake angle, helix angle, drill diameter, cutting speed, feed rate, and depth of cut. The membership functions VL, L, M, H, and VH stand for very low, low, medium, high, and very high, respectively. The sets for the cutting parameters consist of five members whereas the others consist of only three because the effects of the former are more comprehensively investigated by the researchers. The members of the fuzzy sets associated with helix angle and the three cutting parameters are not evenly distributed. The lower ranges of the parameters are covered with more members because they are more widely utilized, in research as well as industry, than the higher ones.

### 3.2. IF-THEN Rules

The heart of the sustainability estimation system is its knowledge-based that consists of a series of IF-THEN rules. Each of the rules relates a level of a categorical machining parameter or a membership function of a numeric machining parameter with the most suitable membership functions of the sustainability measures’ impact fuzzy set (in Figure 1). The selection of the impact fuzzy set’s most appropriate membership function is based on the “generalized impact” of the parameter’s given level on the sustainability measure. As described before, the generalized impact is obtained by amalgamating the various findings, as reported in different publications, regarding the effects of the given machining parameter on the sustainability measures. Likewise, the antecedent-consequent mapping is completed for all the machining parameters to obtain an all-inclusive rule-base. Table 2 presents the fuzzy rule-base.

The 43 rules presented in the table consist of a single condition in their antecedent parts. Some of the antecedent parts contain an ampersand (&) sign between the two membership functions. It is a logical AND operator that returns the intersection of the two neighboring membership functions of a fuzzy set. For the sake of description, Rule 9 can be read as follows:

IF material hardness is *H* (high) THEN tool acquisition economy is *N* (neutral) AND tool life is *VA* (very adverse) AND productivity is *VA* AND fluid consumption economy is *A* (adverse) AND surface quality is *VF* (very favorable) AND electricity conservation is *A* AND specific energy saving is *VA* AND swarf avoidance has *no effect* AND landfill avoidance (tool) is *EA* (extremely adverse) AND landfill avoidance (workpiece) is *F* AND health has *no effect* AND safety is *N&A* (intersection of neutral and adverse) AND working hours normality is *A*.

For high hardness of a work material, the tool acquisition economy is neutral because the choice of the tool’s material and substrate is dependent on the work material. Understandably, a harder temper of the work material will degrade the tool life and productivity, thus, the members *VA* are assigned to them against the high level of hardness. Likewise, a more-than-normal consumption of a cutting fluid is expected to keep the working temperature within the viable limits.

Moreover, a higher level of specific cutting energy is consumed to cut a harder temper of the work material, thereby, rendering its sustainability contribution ‘very adverse’. On a positive side, high hardness is known to generate better work surface finish, which also causes a reduction in scrapping and landfilling of the workpiece due to a better compliance to the quality specifications. The sustainability performance regarding tool’s landfill is extremely adverse due to the expected short tool life. Lastly, work material hardness has no effect on safety or health, but its high values may cause long working hours due to the resulting low material removal rates.

Likewise, Rule Number 18 can be stated as follows:

IF tool material is *PcBN* (polycrystalline cubic boron nitride) THEN tool acquisition economy is *VA* (very adverse) AND tool life is *VF* (very favorable) AND productivity is *EA* (extremely favorable) AND fluid consumption economy is *VF* AND surface quality is *VF* AND electricity conservation is *F* (favorable) AND specific energy saving is *VF* AND swarf avoidance is *N* (neutral) AND landfill avoidance (tool) is *VF* AND landfill avoidance (workpiece), health, and safety have *no effect* AND working hours normality is *VF*.

As PcBN is an artificially prepared and expensive material, the associated tool acquisition economy is ‘very adverse’. On the other hand, its exquisite mechanical properties pay-off for its high cost in form of long tool life and high productivity. Moreover, a coolant is seldom required for such a wear-resistant tool material. As its tool life is exceedingly long, PcBN does not fill the land at an alarming rate. Finally, the high rates of material removal resulting from the tool’s superior mechanical properties lead to a highly favorable normality of working hours.

It is important to reveal the logic behind selection of the three work materials (carbon steel, alloy steel, and titanium alloys) for the rule-based system. These are the three commonly used work materials which also pose sustainability issues to the machining industry because of their unfavorable mechanical and chemical properties. Although aluminum alloys, magnesium alloys, wood, and wood composites are also commonly used, they do not carry red flags in respect of sustainability because of their exceptionally good machinability characteristics.

Besides the 43 rules presented in Table 2, an additional five “high-priority” rules are presented in Table 3. The antecedent parts of the rules consist of two conditions connected with a logical ‘And’. A fulfillment of the conditions fires the rule and, at the same time, prevents the single-condition rules (listed in Table 2) from firing which include the same machining parameters in their antecedent parts. Therefore, these double-condition rules are labelled as high-priority. The machining conditions covered by the antecedent parts of the high-priority rules constitute special cases which are not represented appropriately or comprehensively by the single-condition rules dealing with the same control parameters. The knowledge encompassed by the high-priority rules has also come from the literature mining.

For instance, the high-priority Rule Number 1 (Table 3) is a replacement of the single-condition Rule Numbers 2 and 18 (Table 2). It can be seen that the values in respect of the measures: tool life, productivity, surface quality, landfill avoidance (tool), landfill avoidance (workpiece), and working-hours normality for the high-priority rule are entirely different from those of the two single-condition rules. Technically speaking, PcBN, being an extremely hard and wear-resistant material, is an ideal choice for a continuous cutting process, such as turning, where it yields high productivity and long tool life, avoids coolant’s consumption, and more. On the other hand, its extreme brittleness renders it an unproductive tool material for an interrupted cutting process, such as milling. Repeated engagements and disengagements of the tool’s cutting edges with the work material, in a milling process, require high toughness from the tool material. As high toughness is lacking in PcBN, it becomes an unsuitable tool material for the milling process. Therefore, if used, it would cause an extremely short tool life, low productivity levels, a poor surface finish, and so on. In this context, it can be safely stated that multiple condition rules can evaluate the sustainability measures more aptly that the single-condition rules covering the same control parameters.

### 3.3. Working of the Rule-Base

Firstly, the user is prompted to provide the inputs in respect of all the control parameters of a machining process. The inputs related to the numeric control parameters are fuzzified according to the relevant fuzzy sets provided in Figure 2, whereas the crisp values of the categorical parameters meet no treatment. Next, the conditions in the antecedents of the high-priority rules are checked against the input data (both fuzzified and crisp). A high-priority rule is fired if the conditions are met, and the corresponding single-condition rules are disabled. After going through all the high-priority rules, the antecedents of all the nondisabled single-condition rules are examined for possible matches. All the rules whose conditions are met, are fired. The firing of multiple rules (single-condition as well as double-condition) generate several outcomes in form of fuzzy values. All the fuzzy values of a sustainability measure, generated by firing of different rules, need to be aggregated to achieve its most appropriate estimation. The aggregation of the fuzzy outcomes of a measure are performed using the max–min fuzzy inference method, whose details can be read from the articles [25,26]. The aggregation method returns the measure’s estimation in form of a fuzzy distribution. The center of gravity (CoG) method is utilized to convert the fuzzy distribution into a single numeric value. CoG defuzzifies a fuzzy distribution to a crisp value by returning the centroid of the distribution. In the same way, the crisp estimations are obtained for all the other sustainability measures. The individual scores of the three dimensions of sustainability are then obtained by following the shares’ distribution provided in Table 1. Finally, the sustainability score of the given process is obtained by applying the formula provided in Equation (1).

## 4. Application Examples

The section provides the examples concerning estimation of sustainability for each of the three major machining processes, namely turning, milling, and drilling.

### 4.1. Turning

Turning is a continuous machining process in which a single point cutting tool removes material from the surface of a workpiece (usually in the shape of a solid of revolution) in form of a chip. The workpiece is rotated around its axis whereas the cutting tool is moved linearly/curvilinearly after being fed into the workpiece. The surface speed of the workpiece’s rotational movement is called as cutting speed (m/min) whereas the linear distance covered by the tool per rotation of the workpiece is called as feed rate (mm/rev). Moreover, the depth of cut (mm) is the perpendicular distance measured from the machined surface to the uncut surface of the workpiece.

A rod of an alloy steel, possessing surface hardness of 59 HRc, needs to be turned using a PcBN turning insert (rake angle = 0°) under the effects of emulsion cooling. The cutting speed, feed rate, and depth of cut are fixed to the values of 150 m/min, 0.07 mm/rev, and 0.4 mm, respectively.

The given numeric values of work material hardness, insert’s rake angle, and the three cutting parameters are fuzzified according to their corresponding fuzzy sets. Based on the fuzzified values of the numeric parameters and the crisp values of the categorical ones, Rule Numbers 1, 5, 8 (5% weightage), 9 (95% weightage), 11, 18, 22 (50% weightage), 23 (50% weightage), 26 (60% weightage), 27 (40% weightage), 31 (33% weightage), 32 (67% weightage), 35 (33% weightage), and 36 (67% weightage), as listed in Table 2, are fired. Based on the given conditions, none of the high-priority rules are fired. The numbers of the rules followed by parentheses are those related to the numeric control parameters. The percentage values presented inside the parentheses represent the share by which the corresponding membership function of the fuzzy set relates to the given value of the parameter. The processing of the fuzzy rule-base yields the results regarding sustainability scores, as shown in Table 4. As the total sustainability score (SS) is less than 50, the process following the given conditions should be considered as unsustainable. The weak contribution has significantly come from the societal dimension (*SS*_soc_ = 32.05) as the application of emulsion-based coolant and a small feed rate have drastically cut into the scores of health and working hours normality, respectively.

A sensitivity analysis is performed on the rule-based system to find out the percentage variation in the *SS* with respect to the percentage changes accommodated in each of the five numeric parameters controlled in this example. The analysis recorded –4.9%, +0.9%, +6.8%, −3%, and +2.3% variations in the *SS* for +10% variations adjusted in surface hardness, rake angle, cutting speed, feed rate, and depth of cut, respectively.

### 4.2. Milling

Milling is an interrupted material removing process in which a rotating multiple cutting-edge tool is fed against the workpiece. The material is removed from the workpiece’s surface in form of discontinuous chips. The surface speed of the rotating tool is called as cutting speed (m/min). The feed rate (mm/rev) is defined as the linear distance covered by the milling tool during its one complete rotation. Alternatively, specifically for milling, feed per tooth is defined as the length of work material that is fed into each cutting tooth as it moves through the work material (mm/tooth). Depth of cut (mm), for an end milling process, is defined as the distance the milling tool is fed into the surface of the work material before commencement of its feed movement. Shoulder milling, on the other hand, is characterized by two depths of cut: axial depth of cut (stepdown) and radial depth of cut (stepover).

A titanium alloy needs to be milled using a coated carbide end mill cutter (helix angle = 45°) under the cooling effects of liquid nitrogen. The surface hardness of the work material is 36 HRc. The cutting speed, feed per tooth, and depth of cut are fixed to the values of 65 m/min, 0.1 mm/tooth, and 0.75 mm, respectively.

The crisp values of all the numeric parameters are fuzzified in accordance with their fuzzy sets. Based on the input values, the following rules (listed in Table 2) are fired: 2, 6, 7 (60% weightage), 8 (40% weightage), 14, 17, 21 (50% weightage), 22 (50% weightage), 27, 32 (50% weightage), 33 (50% weightage), 39 (67% weightage), and 40 (33% weightage). The outcomes regarding the sustainability are presented in Table 5. The sustainability scores of the three dimensions show that the given process is marginally sustainable in respect of all the three aspects. The process has scored high regarding tool life and productivity, thanks to the combination of a lower-than-normal work material hardness and a wear-resistant tool. The overall score of just over 50 affirms the process as marginally sustainable.

The sensitivity analysis performed on the results of this example yielded −3.2%, +4.7%, −2.8%, −4.7%, and +4.1% variations in the *SS* for +10% variations adjusted in surface hardness, helix angle, cutting speed, feed per tooth, and depth of cut, respectively.

### 4.3. Drilling

Cutting of a cylindrical hole in a work piece by feeding in a rotating twist drill is called drilling. Surface speed of the outer periphery of the rotating twist drill is called cutting speed (m/min) whereas the axial distance covered by the drill during its one complete rotation is called as feed rate (mm/rev). Depth of cut, in this case, is commonly known as the twist drill’s radius.

Hole-making is required in a plate of plain carbon steel (hardness = 25 HRc) using a carbide (uncoated) drill bit of 8 mm diameter under the lubrication effects of MQL (minimum quantity of lubrication). The two cutting parameters are fixed as follows: cutting speed = 100 m/min and feed rate = 0.1 mm/rev.

In respect of the given conditions, the following rules are fired (Table 2): 3, 4, 7 (100%), 12, 16, 22 (100%), 27 (100%), 41 (40%), and 42 (60%). The further processing of the rule-base yielded the results listed in Table 6. The given process is labelled as sustainable considering the grand sustainability score (SS) of 61. The process fares very well on the economic front, thanks to a long tool life attributed to the excellent machinability of plain carbon steel.

The sensitivity analysis performed on the results of this example recorded −6.3%, −5.5%, +1.5%, and −3.6% variations in the *SS* for +10% variations accommodated in surface hardness, cutting speed, feed rate, and drill diameter, respectively.

## 5. Discussion

The article addresses a contemporary issue of estimating sustainability levels of processes in the manufacturing industry. Sustainability is commonly seen as a qualitative term which depends on lots of attributes and variables, making it difficult to quantify. From the perspective of manufacturing, many of the control parameters are vague and imprecise, which makes the accurate evaluation of sustainability all the more difficult. The best that can be achieved in this regard is to have an estimation of sustainability by considering the manufacturing technology in a holistic manner. The work presented herein epitomizes the very approach. Fuzzy reasoning is used to relate the control parameters of machining technology with the three dimensions of sustainability in the form of IF-THEN rules. Various arithmetic and fuzzy relationships are worked out to establish a parameters–measures–dimensions–sustainability link. The link can be used to estimate the sustainability score of a machining process by utilizing the data in respect of its major control parameters.

The three application examples (Section 4) have provided the sustainability scores of 47, 51, and 61 for the processes of turning, milling, and drilling, respectively against the given values of their control parameters. As such, the three processes should be considered as unsustainable, marginally sustainable, and sustainable, respectively in respect of their current settings of the parameters. For the first two, the practitioners might have concerns on their low *SS* scores. Considering the unacceptable performances on sustainability, as revealed by the fuzzy rule-based system, the turning and milling processes need to be looked into for the possible areas of improvement. The individual sustainability scores of the three dimensions would lead the practitioners to the measures to be fixed in order to boost the grand sustainability score. The identified problematic measures can be improved by optimizing the control parameters which have significant effects on them. The influential parameters having significant effects on the measures can be found by referring to the fuzzy rule-bases provided in Table 2 and Table 3. The entries EF/VF and EA/VA, respectively, represent highly favorable and adverse effects of the corresponding control parameters on the problematic measures. Likewise, the entries F and A, respectively, indicate significant favorable and adverse effects of the corresponding parameters. The settings of these influential parameters can be so changed to have more favorable effects than adverse ones. In such a way, a careful modification of all the influential control parameters can raise the values of the low-scoring measures and, consequently, the grand sustainability score.

The presented work claims novelty regarding the holistic inclusion of the process’s control parameters and the responses supporting the three dimensions of sustainability. The previous works in this regard have either not considered all the dimensions or included a limited number of control parameters in their models. Deng et al. [20] have developed their intelligent expert system for reduction in consumption of specific energy in a cutting process. Although the authors have claimed a potential of 7–15% reduction in energy consumption, the other important sustainability measures related to resources and environment are not included. Likewise, Xu et al. [21] have applied their intelligent reasoning system for estimating energy consumption and optimizing the cutting parameters for enhancing stability of a milling process. Other major aspects of sustainable machining, such as process cost, operational safety, and waste generation are not included. Dhanalakshmi and Rameshbabu, on the other hand, have incorporated environmental and economic attributes in their sustainability assessment model but, yet the social dimension is again neglected [18].

The presented approach is expected to find applicability in the manufacturing sector from the perspective of approximating the sustainability levels of processes/technologies and identifying and fixing the counteracting parameters in case of under par sustainability scores. As the presented system is designed to include all the forms of machining processes for estimation of sustainability in a holistic manner, some control parameters specific to a particular form of machining cannot be accounted for. Therefore, the system is insensitive to the variations in the second-tier control parameters specific to that form of machining process. With a wide-range adoption of sustainable manufacturing in sight, it is recommended to explore other artificial intelligence tools, such as artificial neural network, and metaheuristic algorithms for estimation of manufacturing sustainability and optimization of the process for its maximization. Moreover, explicit sustainability estimation systems can also be worked out for each form of machining along with an in-depth inclusion of all the general as well as specific control parameters.

## Figures and Tables

**Table 1:** The Sustainability Measures of Machining Grouped Under the Three Dimensions of Sustainability.

Economy		Environment		Society	
Measure	Share	Measure	Share	Measure	Share
Tool acquisition economy	25%	Specific energy saving	30%	Health	30%
Tool life	20%	Swarf avoidance	30%	Safety	40%
Productivity	25%	Landfill avoidance (tool)	20%	Working hours normality	30%
Fluid consumption economy	15%	Landfill avoidance (work)	20%	-	
Surface quality	10%	-		-	
Electricity conservation	5%	-		-	

**Table 2:** The Fuzzy Rule-Base for the Estimation of Machining Sustainability.

S/No	Parameter	Level	Economic Measures *						Environmental Measures †				Societal Measures ‡		
			TAE	TL	P	FCE	SQ	EC	SES	SA	L(T)	L(W)	H	S	WN
1	Process	Turning	F	F	N	A	VF	VF	F	F	F	VF	-	N	-
2		Milling	A	N	F	A	A	A	A	A	A	N	-	N	-
3		Drilling	A	N	VF	F	F	N	A	F	N	F	-	N	-
4	Work material	Carbon steel	EF	EF	VF	A	N	VF	VF	-	VF	A	-	-	EF
5		Alloy steel	A	F	N	F	VF	A	A	-	F	F	-	-	A
6		Titanium alloy	VA	A	N	N	F	A	A	-	A	N	-	-	VA
7	Material hardness	L	N	VF	VF	F	VA	F	VF	-	EF	A	-	N & F	F
8		M	N	N	N	N	N	N	N	-	N	N	-	N	N
9		H	N	VA	VA	A	VF	A	VA	-	EA	F	-	N & A	A
10	Cutting fluid	Dry	-	EA	A	EF	VA	EF	N	F	VA	A	F	F	A
11		Emulsion	-	F	F	A	N	VA	A	EA	N	F	EA	N	N
12		MQL	-	F	F	N	EF	N	VF	A	VF	EF	F	N	N
13		Air	-	A	N	F	N	A	F	N	N	N	N	N	N
14		LN_2_	-	VF	F	EA	F	EA	EA	N	VF	F	N	A	F
15		CO_2_	-	VF	F	A	VF	VA	VA	N	VF	F	A	A	F
16	Tool material	Carbide	F	F	F	N	VF	N	F	N	F	-	-	-	N
17		Coated carbide	N	F	VF	F	VF	N	F	N	F	-	-	-	F
18		PcBN	VA	VF	EF	VF	VF	F	VF	N	VF	-	-	-	VF
19		HSS	EF	EA	VA	VA	A	A	A	N	EA	-	-	-	A
20	Cutting speed	VL	N	VF	EA	EA	VA	EA	EA	N	VF	VA	-	N	VA
21		L	N	F	VA & A	VA & A	A	VA & A	A	N	F	A	-	N	A
22		M	N	N	N	N	F	F	F	N	N	VF	-	N	N
23		H	N	A	VF & F	VF & F	VF	F	F	N	A	EF	-	A	F
24		VH	N	EA	EF	EF	N	VF	VF	N	VA	F	-	VA	VF
25	Feed rate	VL	N	N	EA	EA	VF	EA & VA	EA	N	N	F	-	N	VA
26		L	N	F	VA & A	VA & A	EF	A	A	N	F	VF	-	N	A
27		M	N	N	N	N	F	N	N	N	N	F	-	N	N
28		H	N	A	VF & F	VF & F	A	F	F	N	A	A	-	A	F
29		VH	N	EA	EF	EF	EA	VF	VF	N	EA	VA	-	VA & A	VF
30	Depth of cut	VL	N	F	EA	EA	F	EA & VA	EA	A	F	F	-	N	VA
31		L	N	N & F	VA & A	VA & A	N & F	A	A	N	N & F	N & F	-	N	A
32		M	N	N	N	N	N	N	N	F	N	N	-	N	N
33		H	N	A	VF & F	VF & F	A	F	F	N	N & A	A	-	A & N	F
34		VH	N	VA	EF	EF	EA	VF	VF	A	A & VA	VA	-	A	VF
35	Rake angle (*Process = Turning*)	L	N	F	N	N	A	A	A	N	F & N	A & N	-	-	-
36		M	N	N	N	N	N	N	N	N	N	N	-	-	-
37		H	N	A	N	N	F	F	F	N	N & A	N & F	-	-	-
38	Helix angle (*Process = Milling*)	L	N	A	N	N	A	A	A	N	A & N	A	-	-	-
39		M	N	F	N	N	F	A & N	A & N	N	F & N	F	-	-	-
40		H	N	N	N	N	N & F	N	N	N	N	N & F	-	-	-
41	Drill diameter (*Process = Drilling*)	L	VF		A	A	-	F	N	-	N	F	-	F	F
42		M	N		N	N	-	N & A	A	-	A & VA	N	-	N	N
43		H	VA		F	F	-	VA	VA	-	EA	A	-	A	A

* TAE, TL, P, FCE, SQ, and EC stand for tool acquisition economy, tool life, productivity, fluid consumption economy, surface quality, and electricity conservation, respectively; † SES, SA, L(T), and L(W) stand for specific energy saving, swarf avoidance, landfill avoidance (tool), and landfill avoidance (workpiece), respectively; ‡ H, S, and WN stand for health, safety, and working-hours normality, respectively.

**Table 3:** The “High-Priority” Fuzzy Rules *.

S/No	Antecedents	Consequents												
		TAE	TL	P	FCE	SQ	EC	SES	SA	L(T)	L(W)	H	S	WN
1	Process is Milling AND Tool Material is PcBN	VA	EA	A	N	VA	A	A	N	EA	A	-	A	A
2	Work material is Titanium AND Tool material is PcBN	VA	F	F	VF	N	N	N	N	F	N	-	N	F
3	Work material is Carbon Steel AND Cutting Fluid is (LN_2_ or CO_2_)	EF	N	F	VF	A	VA	A	N	F	N & A	N & A	A	N & F
4	Process is Drilling AND Cutting Fluid is Air	A	A & VA	N & A	N&F	A	VA	VA	N & A	N	N	N	A	N
5	Work material is Titanium AND Cutting Speed is VH	VA	EA	EF	EF	VA	VA	VA	N	EA	A	-	VA	VF

* See the footnote of Table 2 for the complete terms represented by the acronyms used herein.

**Table 4:** The *SS* Results of the Example Related to the Turning Process.

Economic Measures						Environmental Measures				Societal Measures		
TAE	TL	P	FCE	SQ	EC	SES	SA	L(T)	L(W)	H	S	WN
46.3	57.8	52.8	50.2	69.2	47.1	43.0	46.7	47.7	62.9	5.0	47.5	38.5
				*SS*_eco_ =	53.14			*SS*_env_ =	49.01		*SS*_soc_ =	32.05
					*SS* =	47.27						

**Table 5:** The *SS* results of the example related to the milling process.

Economic Measures						Environmental Measures				Societal Measures		
TAE	TL	P	FCE	SQ	EC	SES	SA	L(T)	L(W)	H	S	WN
44.4	56.5	58.7	46.0	55.3	41.0	44.8	48.9	55.6	55.1	50.0	47.6	51.3
				*SS*_eco_ =	51.53			*SS*_env_ =	50.26		*SS*_soc_ =	49.44
					*SS* =	50.6						

**Table 6:** The *SS* results of the example related to the drilling process.

Economic Measures						Environmental Measures				Societal Measures		
TAE	TL	P	FCE	SQ	EC	SES	SA	L(T)	L(W)	H	S	WN
58.9	77.1	64.3	51.1	63.6	64.5	62.0	50.0	63.9	62.3	65.0	55.6	62.9
				*SS*_eco_ =	63.46			*SS*_env_ =	58.84		*SS*_soc_ =	60.59
					*SS* =	61.04						

## Data Availability

Not applicable.
